# Supplementary figures and images for: Genome-wide SNP analysis to assess the genetic population structure and diversity of Acrocomia species
Source: PLoS One. 2021 Jul 20;16(7):e0241025. doi: 10.1371/journal.pone.0241025 (PMC8291712; doi:10.1371/journal.pone.0241025)

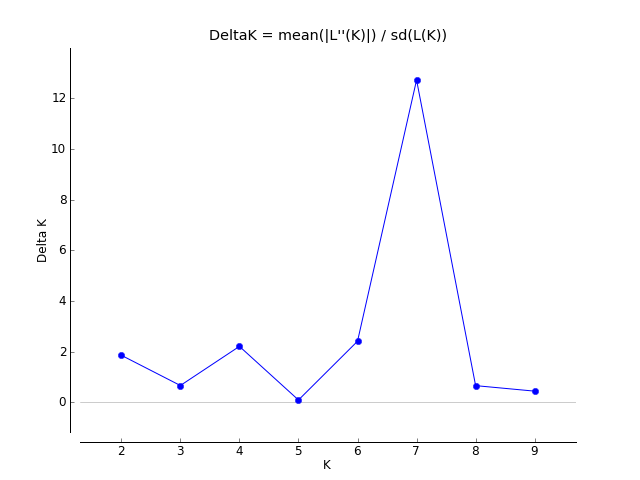

Supplement: S1 Fig — (PNG) [file pone.0241025.s001.png]

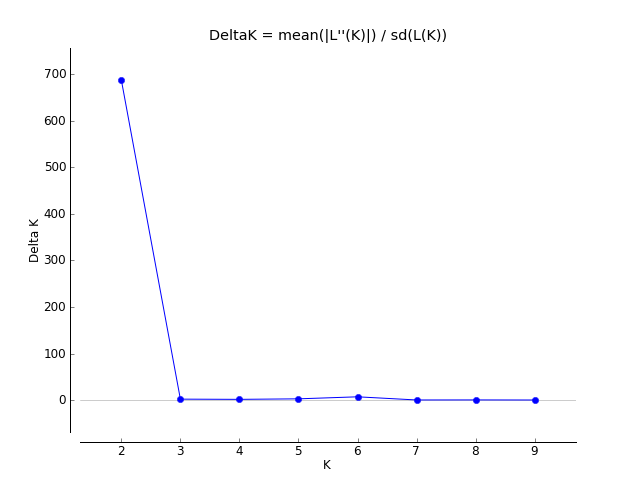

Supplement: S2 Fig — (PNG) [file pone.0241025.s002.png]

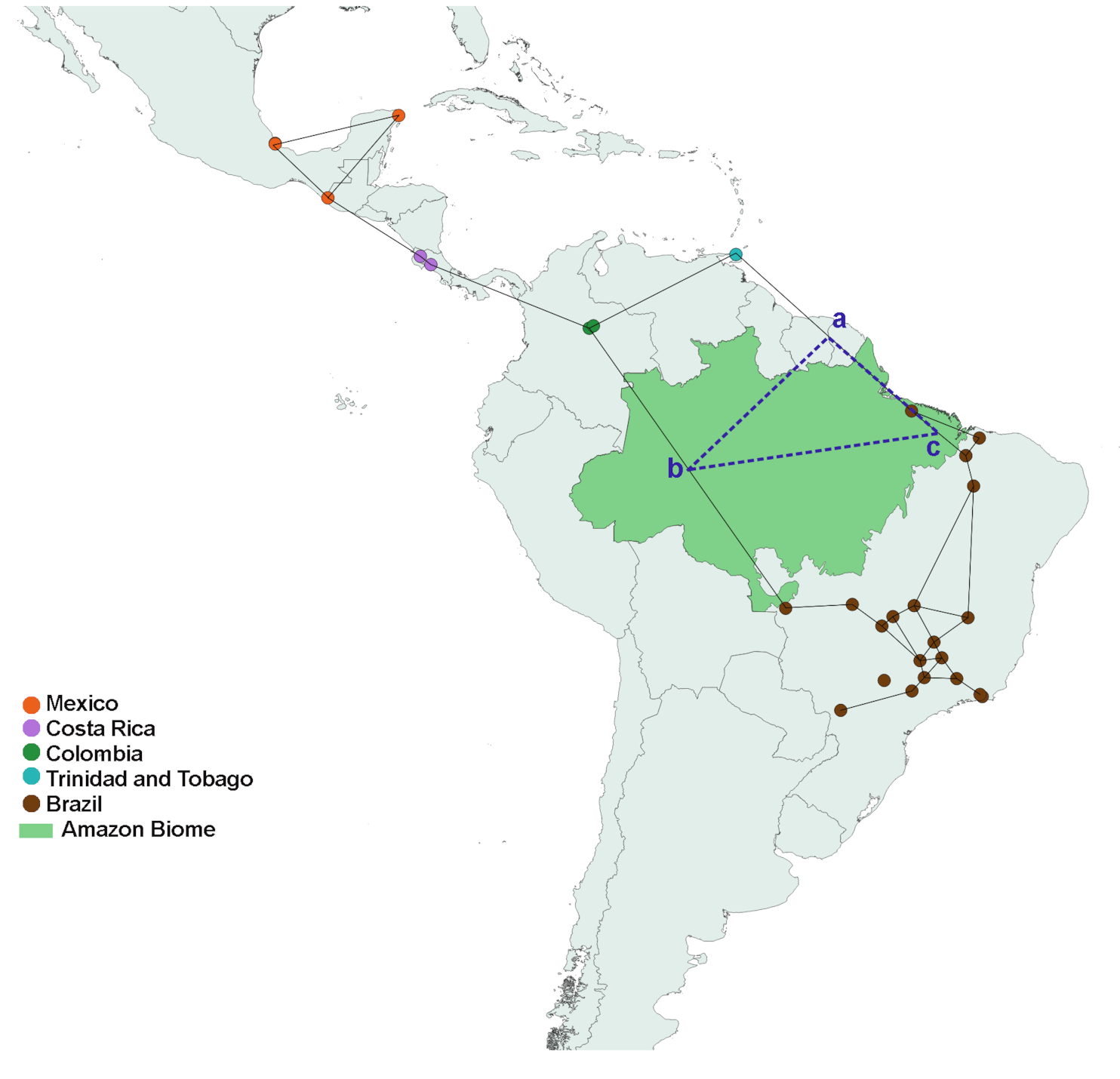

Supplement: S3 Fig — The blue lines indicate the position of the barriers. (TIF) [file pone.0241025.s003.tif]
