# Supplementary material for: Genome-wide SNP analysis to assess the genetic population structure and diversity of Acrocomia species
Source: PLoS One. 2021 Jul 20;16(7):e0241025. doi: 10.1371/journal.pone.0241025 (PMC8291712; doi:10.1371/journal.pone.0241025)
Supplement: S2 File — (DOCX) [file pone.0241025.s008.docx]

**S2 File. Additional information of methods used for outlier SNP identification**

We identified neutral SNPs and loci putatively under selection (outliers). To reduce the possibility of identifying false positives, we applied three approaches to identify outlier loci. For the first approach, we used the method based on Principal Component Analysis (PCA) from the *pcadapt* package [46], on the R platform [47]. The pcadapt method assumes that SNPs excessively related to the population structure are candidates to be under adaptive selection. In this approach, no a priori information about the number of populations was introduced. Initially, we carried out the principal component analysis (PCA) to define the structure of the data set, adopting the Mahalanobis distance from the z-scores in the first k-components of each locus to identify those most related to the population structure. In the second approach, we used the *fsthet* package [48] based on Wright's FST fixation index [49] to identify the loci with deviation from the expected relationship between FST and heterozygosity (HE), using the island migration model [50].

The third approach we adopted to test the association of environmental variables with the genetic variation of SNP markers was the LFMM (Latent Factor Mixed Models) [51], using the LEA package (Landscape Genomics and Ecological Association Test) [52] on R platform [47]. We used nineteen bioclimatic variables related to precipitation and temperature, in addition to the minimum, average and maximum values of wind speed, vapor pressure, and solar radiation, obtained from the WorldClim database [53]. We performed the analyses with the following variables (correlation ≤ 0.8): average annual temperature, average daytime variation, isothermality, average temperature of the wettest four-month period, annual temperature variation, annual precipitation, precipitation in the driest month, precipitation seasonality, radiation maximum solar radiation, minimum solar radiation, and average wind speed. For the lfmm function, five replicates were performed with 200,000 MCMC interactions after 50,000 burn-ins. For the association tests, the genetic structure presented between the individuals was considered with the SNMF analysis [51], determining the most likely number of genetic groups for the different data sets, using 100,000 MCMC interactions, and 10 repetitions⁠ for the number of groups (K) varying between 1 to 15. The LFMM analysis considered K = 8 (Acrocomia species) and 6 (*A. aculeata*). The p-values were adjusted using the genomic inflation factor (λ) and the false discovery rates (FDR) were defined using the Benjamini-Hochberg algorithm, considering FDR = 0.1.

The identification of SNPs hypothetically under selection (outliers) was performed independently for the following groups: 1) In the genus Acrocomia, considering the species as groups, and 2) within *A. aculeata*, considering as groups the samples' countries of origin. We considered as loci putatively under selection those shared between the three identification methods (fsthet, pcadapt and LFMM) (S2 Table). Consequently, we adopted the remaining SNPs considered neutral for the analysis of population genomic diversity and structure.
